# HEALTHY LIFE EXPECTANCY OF OLDER HISPANICS: THE INFLUENCE OF NEIGHBORHOOD CHARACTERISTICS

**DOI:** 10.1093/geroni/igz038.2625

**Published:** 2019-11-08

**Authors:** Kerstin G Emerson, Anqi Pan, Hanwen Huang, Kyriakos Markides

**Affiliations:** 1 University of Georgia, Athens, Georgia, United States; 2 University of Texas Medical Branch, Galveston, Texas, United States

## Abstract

Research consistently shows a survival advantage among Hispanics, despite a worse health profile. The goal of this study was to calculate disability free life expectancy for older Hispanics in the United States, and to explore any difference by neighborhoods. We used data from the Wave 5 (2004-5) of the Hispanic Established Population for the Epidemiological Study of the Elderly (Hispanic EPESE), linked to vital status data through 2016. We used Sullivan’s method to create disability free life expectancy (DFLE) estimates, and to calculate the ratio of life expectancy without disability to life expectancy with disability. These estimates were compared across neighborhood characteristics using Census FIPS data. All neighborhood characteristics were cut into tertiles and significance testing compared high versus low. The average age of the sample was 82 (range 75-109), a majority female (62%), non-married (57.5%), and born in the US (56%). Results showed that neighborhood Hispanic density, poverty, and percent linguistically isolated were not statistically significant for disability free life expectancy estimates. However, disability free life expectancy was higher in neighborhoods with higher density of immigrants, compared to neighborhoods with lower density of immigrants. This was statistically significant for all age groups 75 until age 88. These results suggest that for very old Mexican Americans living in the southwest, neighborhood effects are not significant predictors of disability free life expectancy. The exception is for the neighborhood immigrant density. This suggests that the healthy immigrant effect may maintain even in older ages. Policy and practice implications will be discussed.

